# Deep learning in differentiating the colorectal cancer combined with hepatic enhancing nodules: liver metastases *vs* hemangiomas

**DOI:** 10.1186/s13244-025-02192-2

**Published:** 2026-01-26

**Authors:** Shenglin Li, Shanshan Zhang, Yuebo Wang, Ting Lu, Xinmei Yang, Jialiang Ren, Zhimei Jiao, Yaqiong Ma, Yuan Xu, Yufeng Li, Long Yuan, Yu Guo, Haisheng Wang, Fengyu Zhou, Qianqian Chen, Jianqiang Liu, Junlin Zhou, Guojin Zhang

**Affiliations:** 1 Department of Radiology, Sichuan Provincial People’s Hospital, University of Electronic Science and Technology of China, Chengdu, China; 2 Department of Evidence-Based Medicine and Social Medicine, School of Public Health, Chengdu Medical College, Chengdu, China; 3 Department of Radiology, Lanzhou University Second Hospital, Lanzhou, China; 4 Second clinical school, Lanzhou University, Lanzhou, China; 5 Key Laboratory of Medical Imaging of Gansu Province, Lanzhou, China; 6 Gansu International Scientific and Technological Cooperation Base of Medical Imaging Artificial Intelligence, Lanzhou, China; 7 Department of Pharmaceuticals Diagnostics, GE HealthCare, Beijing, China; 8 Department of Medical Imaging, Zhangye People’s Hospital Affiliated to Hexi University, Zhangye, China; 9 Department of Radiology, Gansu Province Hospital, Lanzhou, China

**Keywords:** Deep learning, Colorectal cancer, Liver metastasis, Hemangiomas, Tomography (X-ray computed)

## Abstract

**Objectives:**

To assess a deep learning (DL) model using portal-venous phase CT for discriminating colorectal cancer liver metastasis (CRLMs) and hemangiomas (HMs).

**Materials and methods:**

Colorectal cancer (CRC) patients diagnosed with CRLMs or HMs at two medical centers from January 2018 and April 2024 were retrospectively included. Lesions were automatically segmented using TotalSegmentator. DL models, DenseNet-201 and ResNet-152, were trained to classify CRLMs and HMs. Their performance, measured by AUC, was evaluated on validation and test sets. Subgroup analyses were conducted for lesions ≤ 10 mm (subcentimeter) and 10–30 mm. Radiologists’ diagnostic performance with and without DL assistance was compared using a multi-reader multi-case analysis.

**Results:**

534 CRLMs (134 CRC-patients; median, 60 years) and 262 HMs (154 CRC-patients; median, 62 years) were divided into the training, validation and test set. The Dice coefficients of TotalSegmentor for automatically segmenting subcentimeter and 10–30 mm lesions were 0.692 ± 0.099 and 0.861 ± 0.033, respectively (*p* < 0.01). ResNet-152 model achieved AUCs of 0.875 (95% CI: 0.838–0.912), 0.858 (95% CI: 0.781–0.935), 0.776 (95% CI: 0.703–0.848) for classifying CRLMs and HMs on the training, validation, and test sets, respectively. The AUCs for distinguishing between 10–30 mm CRLMs and HMs improved from 0.851 (95% CI: 0.821–0.880) to 0.879 (95% CI: 0.853–0.906) with DL assistance compared to without (*p* = 0.015). For subcentimeter CRLMs and HMs, the AUCs for the radiologists and the DL-assisted diagnosis were 0.742 (95% CI: 0.669–0.814) and 0.763 (95% CI: 0.681–0.845), respectively (*p* = 0.558).

**Conclusion:**

DL can assist radiologists in distinguishing 10–30 mm CRLMs from HMs in CRC patients. The value of DL-assisted diagnosis is limited for subcentimetre CRLMs and HMs.

**Critical relevance statement:**

Dynamic detection of hypoenhancing liver lesions in patients with CRC is exceptionally challenging. The DL tool we have developed can assist in evaluating CRLMs and HMs.

**Key Points:**

- TotalSegmentator can perform automatic segmentation of CRLMs and HMs, but demonstrates poorer segmentation consistency for subcentimeter lesions.
- This DL model assists radiologists in distinguishing 10–30 mm CRLMs from HMs in CRC patients.
- Subcentimeter CRLMs and HMs can require further MRI scanning.

**Graphical Abstract:**

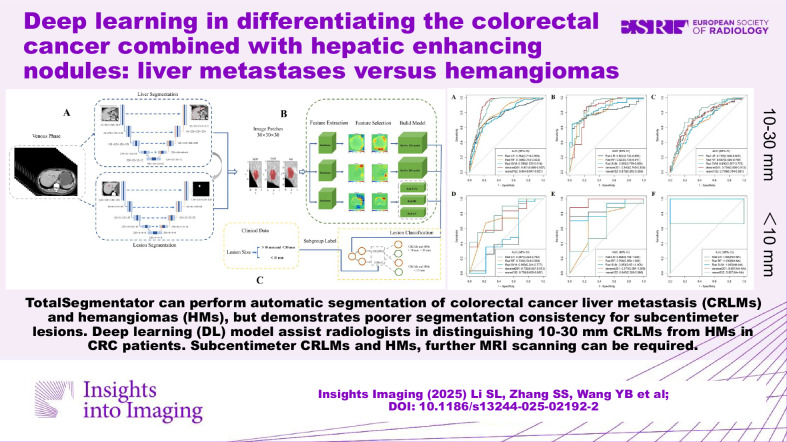

## Introduction

Colorectal cancer (CRC) is a malignancy with both a high incidence and mortality rate globally [1, 2]. Among newly diagnosed CRC, 20% present with metastatic disease at initial diagnosis, while an additional 25% of patients initially staged with localized disease subsequently develop metastatic progression [3]. Liver is the most common site of metastatic spread, and CRC liver metastases (CRLMs) are a major factor affecting patients’ long-term survival [3, 4]. Therefore, prompt detection and characterization of CRLMs through radiological assessment is crucial for selecting the appropriate disease management and treatment plan. However, CRLMs can easily be confused with hepatic hemangiomas (HMs), which are the common benign liver lesions, particularly when lesions are ≤ 10 mm (subcentimeter) [5–10]. This can lead to incorrect metastasis staging and unnecessary treatment for patients during follow-up.

The National Comprehensive Cancer Network (NCCN) and European Society for Medical Oncology recommend contrast-enhanced computed tomography (CECT) imaging as the preferred imaging tool for preoperative evaluation and postoperative follow-up of CRC patients [11, 12]. However, the diagnostic performance of radiologists relying on CECT to differentiate between CRLMs and HMs is generally limited [9]. The polytomous discrimination index for radiologists in differentiating subcentimeter CRLMs and HMs was 0.846–0.939 [9]. Previous studies have shown that gadoxetic acid-enhanced magnetic resonance imaging (MRI) is superior to CECT in detecting and characterising subcentimeter hepatic lesions [13–15]. MRI demonstrated a diagnostic accuracy of 0.965 and sensitivity of 97.47% for subcentimeter liver metastases (LMs) [16]. The diagnostic superiority of MRI notwithstanding, CECT-based evaluation of LMs remains integral to CRC care pathways, particularly given its entrenched role in routine clinical practice. Therefore, further methodological refinement is warranted to enhance diagnostic confidence when characterising indeterminate hepatic lesions with CECT.

Deep learning (DL) assisted medical diagnosis has become an important direction of future medical development. DL based on CECT can enable the early detection and diagnosis of pancreatic cancer and focal liver lesions [17, 18]. Among these, the DL diagnostic tools for focal liver lesions have not performed subgroup analyses based on lesion size, leaving us uncertain about their efficacy in distinguishing subcentimeter CRLMs and HMs [18]. In addition, radiomics is a technology that extracts qualitative data from medical images and is used to support disease detection and diagnosis [19]. Bae et al [9] demonstrated that a radiomics model can effectively differentiate between CRLMs, HMs, and hepatic cysts. However, the performance of the radiomics model is less effective when hepatic lesions are smaller than 10 mm. Whether the differentiation performance of the model can be further optimized through 3D lesion features or DL methods warrants further investigation.

In this study, we aimed to evaluate the performance of the DL model in classifying HMs and CRLMs using portal-venous phase CT images, and to compare its diagnostic accuracy with that of radiologists.

## Materials and methods

The ethics committee of two centers approved this retrospective study and waived the requirement for informed consent (approval numbers: 2022A-298 and 2025-292).

### Datasets and design

**Fig. 1:**
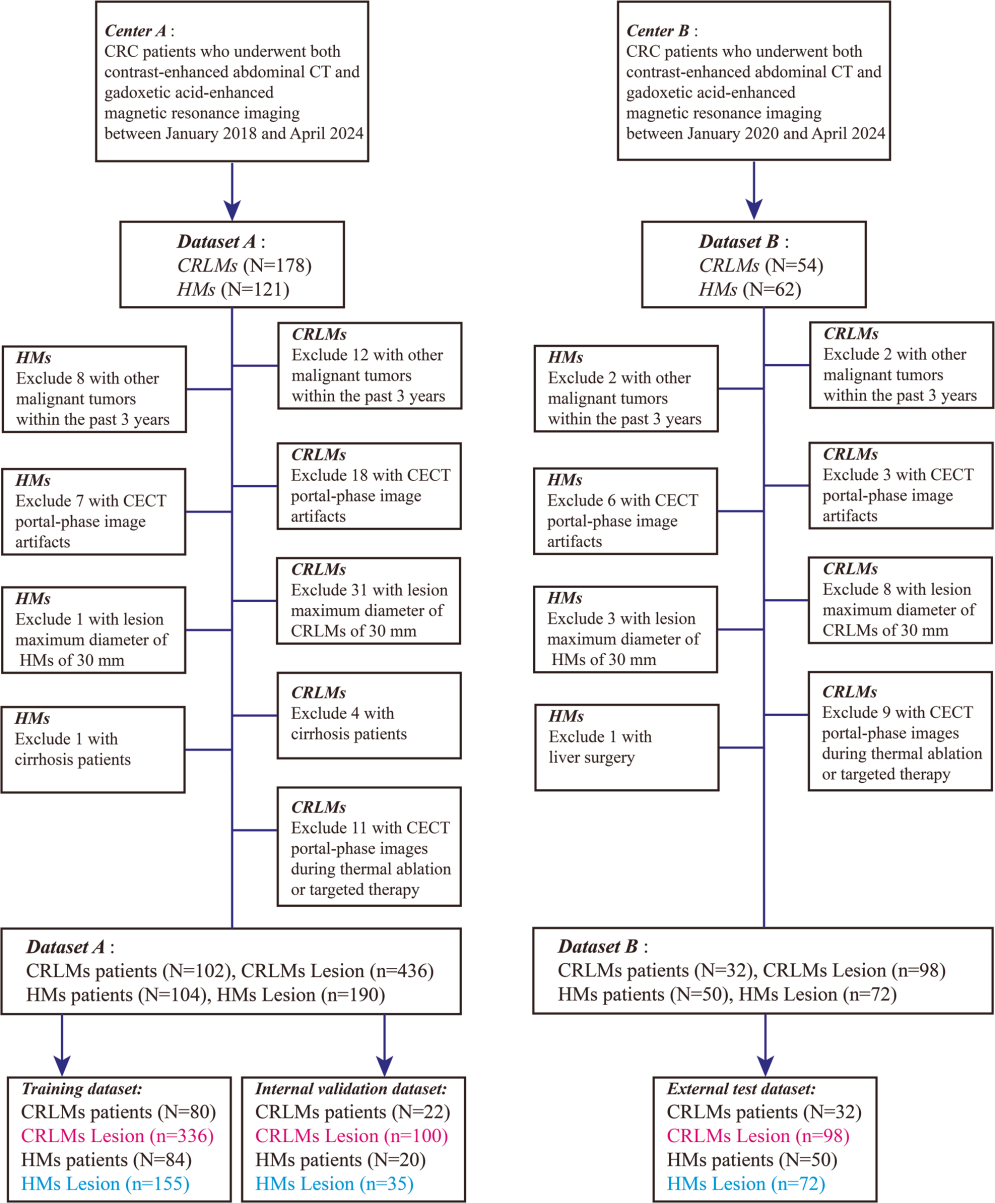
A flow diagram of patient selection. CRC, colorectal cancer; CRLMs, colorectal cancer liver metastasis; HMs, hemangiomas; CT, computed tomography

CRC patients with CRLMs or HMs admitted to two hospitals (Lanzhou University Second Hospital and Sichuan Provincial People’s Hospital) between January 2018 and April 2024 were considered for inclusion in this retrospective study. The inclusion criteria were (a) over 18 years or older, (b) pathology confirmed the CRC, (c) CRLMs and HMs were confirmed either by liver biopsy pathology, gadoxetic acid-enhanced MRI, or through multiple follow-up observations, and (d) liver lesion number less than 10. The exclusion criteria were (a) other malignancies, (b) CECT portal-venous phase image artifacts, (c) all lesions maximum diameter of more than 30 mm or greater, (d) cirrhosis patients, (e) liver surgery, and (f) CECT portal-venous phase images during thermal ablation or targeted therapy (Fig. 1).

The patients’ characteristics, including age, sex, and diameter of lesion (mm), were acquired from electronic database and picture archiving and communication system (PACS) records.

### Lesion detection and reference standards

All lesions with confirmed CRLMs and HMs based on biopsy or conventional cross-sectional imaging techniques [MRI, Positron emission tomography/X-ray computed tomography (PET/CT)] were randomly assigned for clinical assessment. In addition, the reference standards were not applied sequentially. Given the retrospective nature of the study, all lesions diagnosed under the reference standard were corroborated by serial follow-up imaging. Following multidisciplinary team discussion and collective consideration of the reference standards, definitive characterization of hepatic nodules as either CRLMs or HMs was established (Table S1).

### CECT image acquisition and procedures

Three manufacturers' CT scans were used in this study. The CECT scanning parameters are listed in Tables S2 and S3. Image acquisition is described in the Supplementary Material.

### Detection and diagnosis of lesions by radiologists

Portal-venous phase CT images of all 288 CRC patients were retrospectively reviewed. Before the radiologist’s retrospective review of the CECT images, an independent radiologist not involved in the imaging assessment annotated all confirmed lesions according to the reference standard. Five radiologists (H.W., J.L., G.Z., Y.M., and J.Z., with 3, 3, 6, 14 and 35 years of abdominal radiology experience, respectively) with different diagnostic experiences assessed the likelihood that all annotated lesions represented CRLMs or HMs. Classified CRLMs and HMs were independently evaluated by radiologists applying the Lister scoring scale through a single-blind validated protocol. Each lesion was scored for diagnostic confidence (i.e., 1 indicating definitely HMs, 2 indicating probably HMs, 3 indicating indeterminate, 4 indicating probably CRLMs, and 5 indicating definitely CRLMs). There was no time limit for the interpretations, but radiologists were required to review the portal-venous phase CT images in a manner similar to clinical practice.

The evaluated lesions were compared with reference standards (Diagnostic confidence score ≤ 2 was described as HMs; otherwise, it was described as CRLMs). HMs with a diagnostic confidence score ≥ 3 were considered false positive results relative to the reference standard. CRLMs demonstrating diagnostic confidence scores ≤ 2 on the Lister scoring scale were reference standard confirmed as false-negative determinations [20, 21].

### Liver automatic segmentation

Image standardization, segmentation, and post-processing are described in the Supplementary Materials. In the methodological foundation of this investigation, automated hepatic parenchyma segmentation was implemented on CECT datasets utilising TotalSegmentator (accessible at: https://github.com/wasserth/TotalSegmentator), a DL-based segmentation framework originally developed by Wasserthal et al [22] for volumetric quantification of anatomical structures in cross-sectional imaging. This open-source algorithm enables reproducible organ delineation through its validated nnU-Net architecture, serving as the baseline anatomic reference for subsequent lesion characterization. This DL-based software utilises a pre-trained 3D U-Net architecture to identify and delineate the liver parenchyma.

### Lesions segmentation

All diagnosed CRLMs and HMs will be manually 3D segmented using ITK-SNAP software (http://www.itksnap.org/, version 4.2.0). To improve the validity and accuracy, segmentation was performed by five radiologists (S.L., X.Y., TL., Y.L., and Y. X.) with 9, 4, 4, 7 and 7 years of abdominal radiology experience, respectively. Lesion automatic segmentation is described in the Supplementary Materials.

### Conventional radiomic features extraction

The open source “PyRadiomics” package [23] was used to extract traditional radiomic features. Radiomics feature extraction and model construction are described in the Supplementary Material.

### Conventional radiomics signature building

Radiomics feature selection and label construction were performed on the portal-venous phase CT images. Logistic regression (LR) models, random forest (RF) models, and support vector machine (SVM) models were constructed based on the volume of interest (VOI) of the lesions and various machine learning methods, as described in the Supplementary Material.

### DL model construction

Following the automated lesion segmentation, each individual lesion was meticulously cropped from the liver region to facilitate precise analysis and classification. To ensure uniformity and manage computational resources effectively, only lesions with diameters less than 30 mm were retained. The cropping process standardized each lesion to a fixed matrix size of 30 × 30 × 30 voxels, corresponding to a physical dimension of 30 × 30 × 30 mm³, consistent with the prior voxel normalization to 1 × 1 × 1 mm³. This uniform spatial dimension ensured that all input data for the classification models were consistent, thereby enhancing model performance and reliability.

**Fig. 2:**
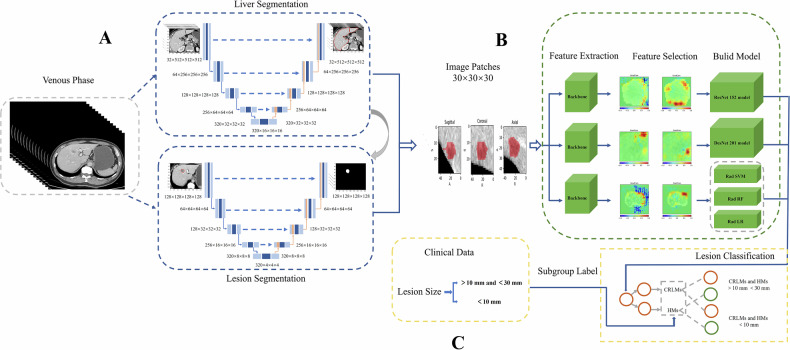
A flowchart of the AI model. **A** Automated segmentation of liver lesions. **B** Feature extraction of CRLMs and HMs. **C** Diagnostic classification and subgroup analysis of CRLMs and HMs

For the classification of lesions into CRLMs or HMs, we employed two advanced three-dimensional convolutional neural network architectures: ResNet3D and DenseNet3D. These models were selected for their robust ability to capture and learn complex spatial features inherent in volumetric medical imaging data. The specific DL model development process is described in the Supplementary Material. A flowchart of this study and the ResNet-152 (RN152), DensNet-201 (DN201), and radiomics (Rad SVM, Rad RF, and Rad LR) modelling pipeline are shown in Fig. 2, respectively.

### Performance evaluation

We used the Dice coefficient, Jaccard coefficient, sensitivity and specificity to evaluate the efficacy of hepatic lesions automatic segmentation. The area under the receiver operating characteristic (ROC) curve (AUC), accuracy (ACC), sensitivity, specificity, positive predictive value (PPV), and negative predictive value (NPV) were used to evaluate the performance of RN152, DN201 and the Radiomics model (LR, RF, SVM model) for distinguishing CRLMs from HMs. The confusion matrix serves as a visual representation of the classification performance of DL models. The DeLong testing method was used to compare the AUC differences between RN152, DN201 and the Radiomics model.

### Statistical analysis

Differences in patients’ radiomics features in the dataset were assessed by an independent *T*-test or Mann–Whitney *U*-test for continuous variables, while for categorical variables, the Fisher exact test or Chi-square test was used. The Kruskal–Wallis *H*-test is used to compare three or more groups. Implementing the multi-reader multi-case analysis based on the analysis of variance (ANOVA) model (‘MRMCaov’ package), to analyze the differences in diagnostic accuracy, sensitivity, and specificity between five readers. The parameters of error, variance, and covariance are calculated by determining the mean values of all readers and specific test estimates, which are in turn obtained through the jackknife method. All tests were two-tailed, and *p* ≤ 0.05 was considered statistically significant. Data were analyzed by two authors (S.Z. and J.R.) using R software, version 4.2.0 (http://www.Rproject.org; R Foundation for Statistical Computing) and Python software, 3.8.0 (https://www.python.org/). The DL model is trained on the NVIDIA RTX4090 24GB.

## Results

### Patient characteristics

The study included 288 consecutive CRC patients (median age, 61 years; IQR, 52–69 years; 169 men) with 134 CRLM patients (median age, 60 years; IQR: 51–68 years; 81 men) and 154 HMs patients (median age, 62 years; IQR: 53–70 years; 88 men). Baseline CRC patient and tumor characteristics of the training, validation and external test set are summarized in Table 1. Hepatic lesions in dataset A were assigned to the training and validation set according to the ratio of 7:3. A total of 534 CRLMs and 262 HMs were divided into the training set (CRLMs = 336, HMs = 155), validation set (CRLMs = 100, HMs = 35), and external test set (CRLMs = 98, HMs = 72) (Fig. 1). Mean size of the liver lesions was 14.76 ± 6.31 mm (CRLMs, 14.52 ± 6.23 mm; HMs, 15.25 ± 6.46 mm). Demographics of patients were not significantly different between the three cohorts (Table 2).

**Table 1:** Patient baseline demographics in the training dataset, internal validation dataset and external test dataset

Parameter	Training dataset (*N* = 164)	Internal validation dataset (*N* = 42)	External test dataset (*N* = 82)	*p* value
Mean age (years)	58.68 ± 11.96	58.64 ± 14.62	62.46 ± 10.63	0.056
Sex				
Men (%)	93/164 (56.71%)	25/42 (59.52%)	51/82 (62.20%)	0.707
Female (%)	71/164 (43.29%)	17/42 (40.48%)	31/82 (37.80%)	
CEA level (ng/mL)	15.55 [4.63, 60.28]	12.05 [3.86, 51.15]	16.63 [4.86, 60.01]	0.363
Primary cancer location				
Colon (%)	91/164 (55.49%)	20/42 (47.62%)	57/82 (69.51%)	0.034
Rectum (%)	73/164 (44.51%)	22/42 (52.38%)	25/82 (30.49%)	
Primary cancer T stage				
T2 (%)	8/164 (4.88%)	1/42 (2.38%)	3/82 (3.66%)	0.099
T3 (%)	74/164 (45.12%)	24/42 (57.14%)	27/82 (32.93%)	
T4 (%)	82/164 (50.00%)	17/42 (40.48%)	52/82 (63.41%)	
Primary cancer N stage				
N0 (%)	23/164 (14.03%)	8/42 (19.05%)	6/82 (7.32%)	0.384
N1 (%)	69/164 (42.07%)	18/42 (42.86%)	36/82 (43.90%)	
N2 (%)	72/164 (43.90%)	16/42 (38.09%)	40/82 (48.78%)	

The tumor depth (T) and nodal status (N) were determined according to the guidelines of the eighth edition of the American Joint Committee on Cancer (AJCC) staging manual

*CEA* carcinoembryonic antigen

**Table 2:** Hepatic lesion confirmation in the training dataset, internal validation dataset and external test dataset

Hepatic lesion confirmation	Training dataset (*N* = 491)	Internal validation dataset (*N* = 135)	External test dataset (*N* = 170)	*p* value
Pathological results	85/491 (17.31%)	31/135 (22.96%)	47/170 (27.65%)	/
CRLMs (%)	78/85 (91.76%)	29/31 (93.55%)	44/47 (93.62%)	
HMs (%)	7/85 (8.24%)	2/31 (6.45%)	3/47 (6.38%)	
Confirmatory imaging	337/491 (68.64%)	88/135 (65.19%)	109/170 (64.12%)	
CRLMs (%)	189/337 (56.08%)	55/88 (62.50%)	40/109 (36.70%)	
HMs (%)	148/337 (43.92%)	33/88 (37.50%)	69/109 (63.30%)	
Follow-up imaging	69/491 (14.05%)	16/135 (11.85%)	14/170 (8.24%)	
CRLMs (%)	69/69 (100%)	16/16 (100%)	14/14 (100%)	
HMs (%)	0/69 (0.0%)	0/16 (0.0%)	0/14 (0.0%)	
Size (mm)	14.56 ± 6.34	13.61 ± 6.03	16.26 ± 6.21	0.001

*CRLMs* colorectal cancer liver metastases, *HMs* hemangiomas

### Automatic segmentation performance

**Fig. 3:**
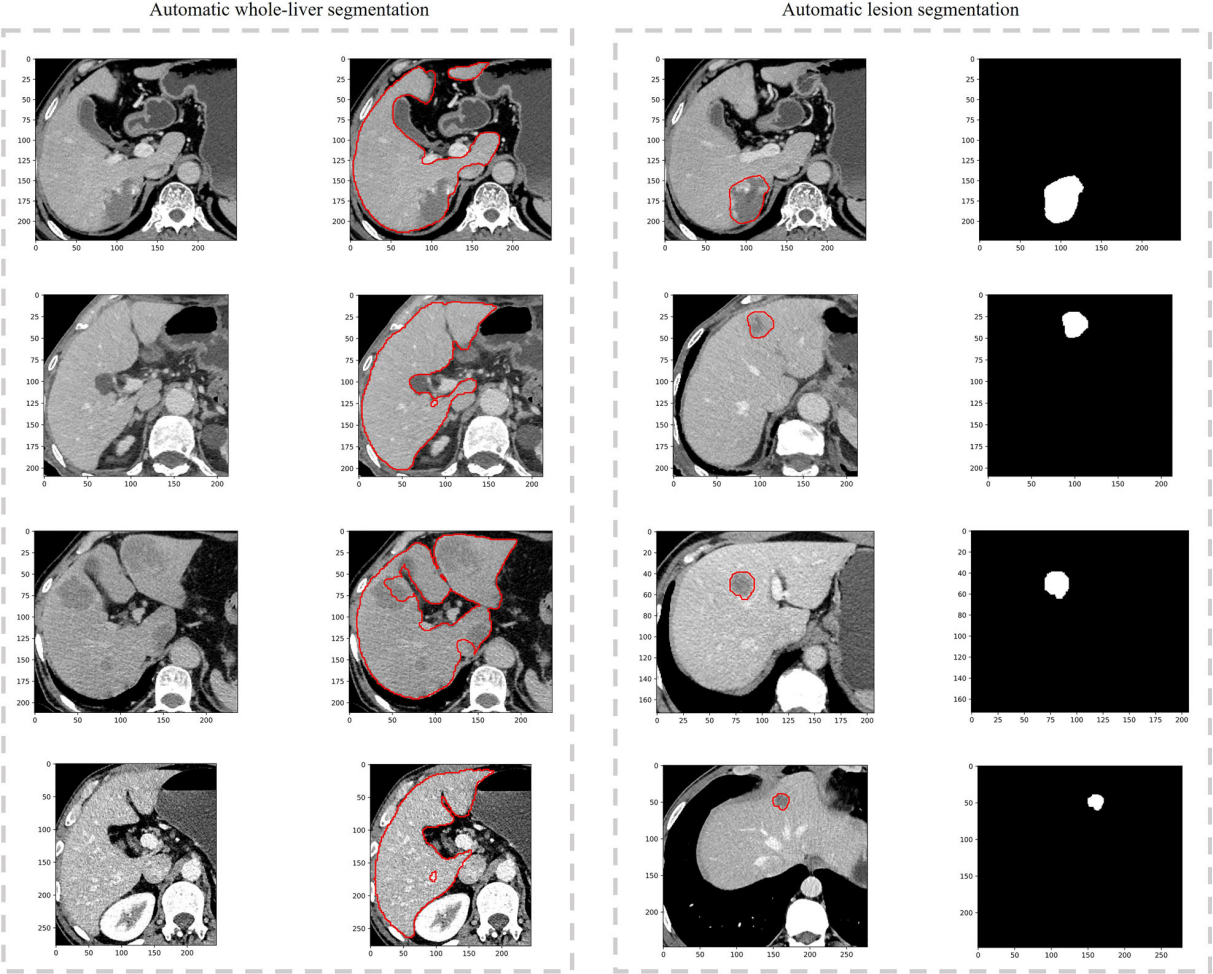
The TotalSegmentator realises the automatic segmentation of the whole liver and lesions

The dice, jaccard, sensitivity, specificity and pred_volume of the TotalSegmentator for automatic segmentation of hepatic lesions were 0.781 ± 0.159, 0.663 ± 0.171, 0.779 ± 0.182, 0.999 ± 0.000, 3006.154 ± 5553.53, respectively (Table 3 and Fig. 3). For subcentimeter lesions, automated segmentation accuracy (Dice: 0.692 ± 0.099) was significantly inferior to that for 10–30 mm lesions (Dice: 0.861 ± 0.033; *p* < 0.01) (Table S4).

**Table 3:** Performance of the TotalSegmentator for automatic segmentation of liver lesions in Dataset A and Dataset B

Group	Dataset A (*N* = 626)	Dataset B (*N* = 170)	*p* Vaule
Calculation			
Dice	0.782 ± 0.161	0.776 ± 0.154	0.683
Jaccard	0.665 ± 0.172	0.655 ± 0.167	0.530
Sensitivity	0.777 ± 0.179	0.786 ± 0.192	0.591
Specificity	0.999 ± 0.000	0.999 ± 0.000	0.606
Labe_volume	2901.578 ± 5707.492	2724.918 ± 3091.559	0.722
Pred_volume	3096.473 ± 6038.882	2656.964 ± 3006.483	0.401

*Labe_volume* manual segmentation volume of liver lesions, *Pred_volume* automatic segmentation volume of liver lesions

### Performance of DL and radiomics in distinguishing CRLMs from HMs

**Fig. 4:**
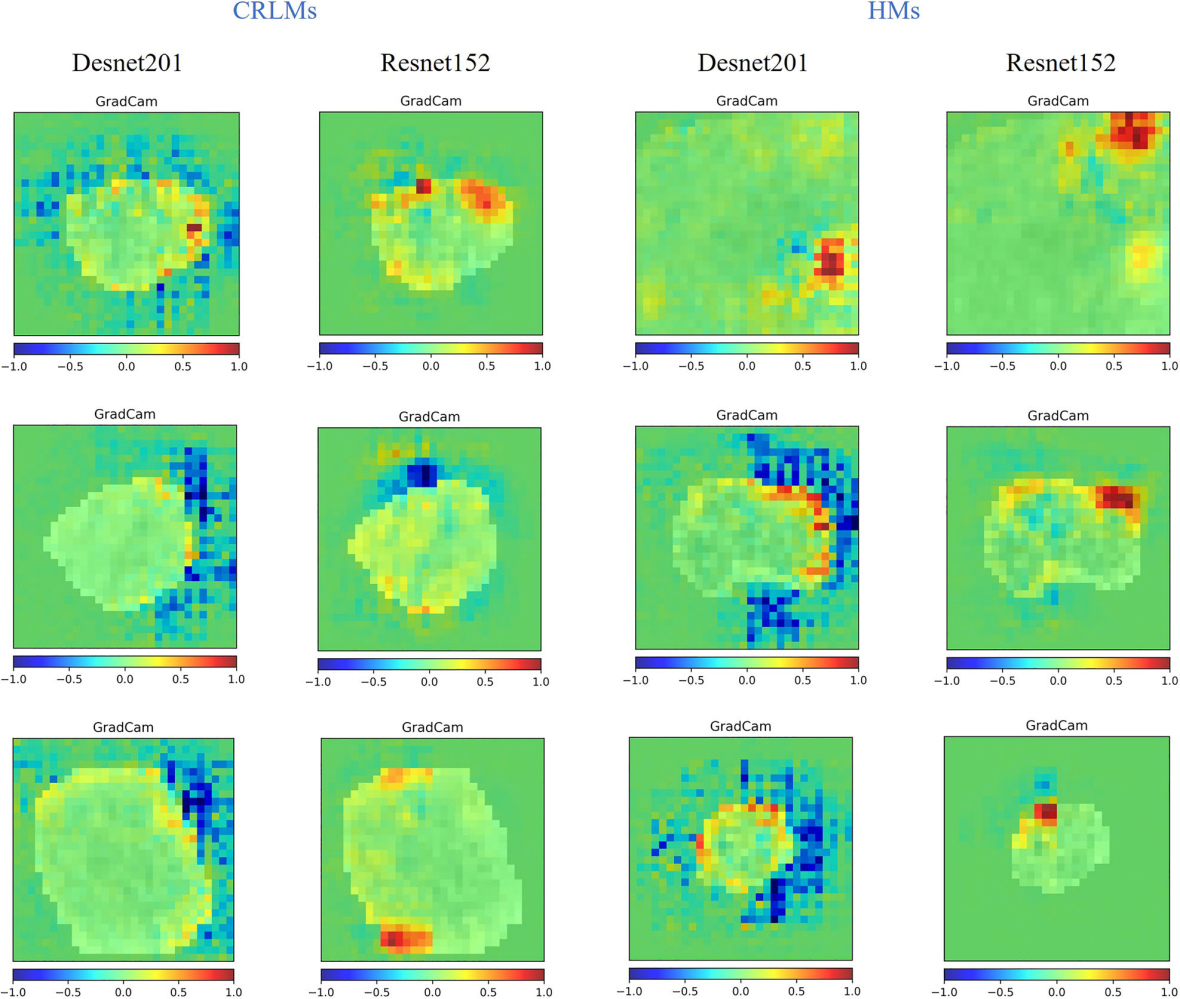
CRLMs and HMs gradient-weighted class activation mapping (GAM). The CAM highlights the class-specific discriminative regions. These heatmaps identify the image regions that most contributed to the classification, effectively showing the model’s reasoning. The models’ focus mirrored radiologists’ diagnostic reasoning, prioritising lesion boundaries and enhancement patterns

All five models showed good discriminatory efficacy in the diagnosis of CRLMs and HMs. Among them, the DN201 and RN152 models have the best diagnostic efficiency (Table 4). The DN201 and RN152 models achieved AUCs of 0.892 (95% CI: 0.857–0.927) and 0.875 (95% CI: 0.838–0.912) in the training set; 0.847 (95% CI: 0.759–0.935) and 0.858 (95% CI: 0.781–0.935) in the validation set; and 0.735 (95% CI: 0.658–0.811), and 0.776 (95% CI: 0.703–0.848) in the test set, respectively, for discriminating CRLMs from HMs. The confusion matrices demonstrate the distribution and nature of misclassifications by the DN201 and RN152 models with respect to distinguishing between CRLMs and HMs (Fig. S1). The Delong test showed that there was no difference in the AUCs between the RN152 and DN201 models in the training, validation and test set (*p* > 0.05). However, there were statistically significant differences compared with the other three radiomics models (*p* ≤ 0.05) (Table S5). The focusing areas of DN201 and RN152 on CECT images were shown in Fig. 4 using Grad-CAM.

**Table 4:** Performance of DL models *vs* radiomics models in distinguishing CRLMs from HMs

Model	Training dataset (*N* = 491)	Internal validation dataset (*N* = 135)	External test dataset (*N* = 170)
Rad LR			
AUC (95% CI)	0.750 (0.704–0.796)	0.814 (0.743–0.885)	0.741 (0.665–0.817)
ACC (95% CI)	0.695 (0.652–0.735)	0.704 (0.619–0.779)	0.676 (0.601–0.746)
Sensitivity (95% CI)	0.652 (0.506–0.732)	0.640 (0.540–0.780)	0.602 (0.388–0.766)
Specificity (95% CI)	0.787 (0.690–0.845)	0.886 (0.743–1.000)	0.778 (0.639–0.861)
PPV (95% CI)	0.869 (0.837–0.882)	0.941 (0.931–0.951)	0.787 (0.704–0.824)
NPV (95% CI)	0.510 (0.478–0.528)	0.463 (0.419–0.493)	0.589 (0.541–0.614)
Rad RF			
AUC (95% CI)	0.784 (0.740–0.829)	0.810 (0.724–0.896)	0.701 (0.622–0.780)
ACC (95% CI)	0.713 (0.671–0.752)	0.711 (0.627–0.786)	0.694 (0.619–0.762)
Sensitivity (95% CI)	0.696 (0.606–0.768)	0.670 (0.390–0.815)	0.643 (0.347–0.725)
Specificity (95% CI)	0.748 (0.665–0.819)	0.829 (0.686–0.971)	0.764 (0.444–0.861)
PPV (95% CI)	0.857 (0.839–0.869)	0.918 (0.867–0.931)	0.787 (0.667–0.807)
NPV (95% CI)	0.532 (0.503–0.555)	0.468 (0.421–0.507)	0.611 (0.478–0.639)
Rad SVM			
AUC (95% CI)	0.756 (0.709–0.802)	0.849 (0.785–0.914)	0.706 (0.627–0.785)
ACC (95% CI)	0.688 (0.645–0.729)	0.733 (0.650–0.806)	0.641 (0.564–0.713)
Sensitivity (95% CI)	0.655 (0.509–0.720)	0.680 (0.570–0.811)	0.571 (0.367–0.735)
Specificity (95% CI)	0.761 (0.658–0.832)	0.886 (0.657–1.000)	0.736 (0.597–0.847)
PPV (95% CI)	0.856 (0.822–0.867)	0.944 (0.934–0.953)	0.747 (0.655–0.791)
NPV (95% CI)	0.504 (0.468–0.527)	0.492 (0.418–0.522)	0.558 (0.506–0.592)
Densnet201			
AUC (95% CI)	0.892 (0.857–0.927)	0.847 (0.759–0.935)	0.735 (0.658–0.811)
ACC (95% CI)	0.855 (0.821–0.885)	0.830 (0.755–0.889)	0.665 (0.588–0.735)
Sensitivity (95% CI)	0.878 (0.681–0.938)	0.850 (0.310–0.950)	0.622 (0.490–0.765)
Specificity (95% CI)	0.806 (0.729–0.865)	0.771 (0.629–0.943)	0.722 (0.597–0.861)
PPV (95% CI)	0.908 (0.884–0.913)	0.914 (0.795–0.922)	0.753 (0.706–0.789)
NPV (95% CI)	0.753 (0.734–0.766)	0.643 (0.595–0.688)	0.584 (0.538–0.626)
Resnet152			
AUC (95% CI)	0.875 (0.838–0.912)	0.858 (0.781–0.935)	0.776 (0.703–0.848)
ACC (95% CI)	0.876 (0.843–0.904)	0.874 (0.806–0.925)	0.741 (0.669–0.805)
Sensitivity (95% CI)	0.961 (0.824–0.997)	0.950 (0.730–1.000)	0.857 (0.694–0.939)
Specificity (95% CI)	0.690 (0.606–0.768)	0.657 (0.429–0.800)	0.583 (0.416–0.667)
PPV (95% CI)	0.871 (0.852–0.875)	0.888 (0.859–0.893)	0.737 (0.694–0.754)
NPV (95% CI)	0.892 (0.879–0.902)	0.821 (0.750–0.848)	0.750 (0.682–0.774)

*AUC* area under the curve, *RF* random forest, *LR* logistic regression, *ACC* accuracy, *PPV* positive predictive value, *NPV* negative predictive value, *Rad* radiomics, *CI* confidence interval

In addition, the assessments of data variability across different CT scanners and models in the training, validation, and test sets are presented in the Supplementary Material (Tables S6–S9). The DL model demonstrated robustness across different scanners.

### Subgroup analysis

**Fig. 5:**
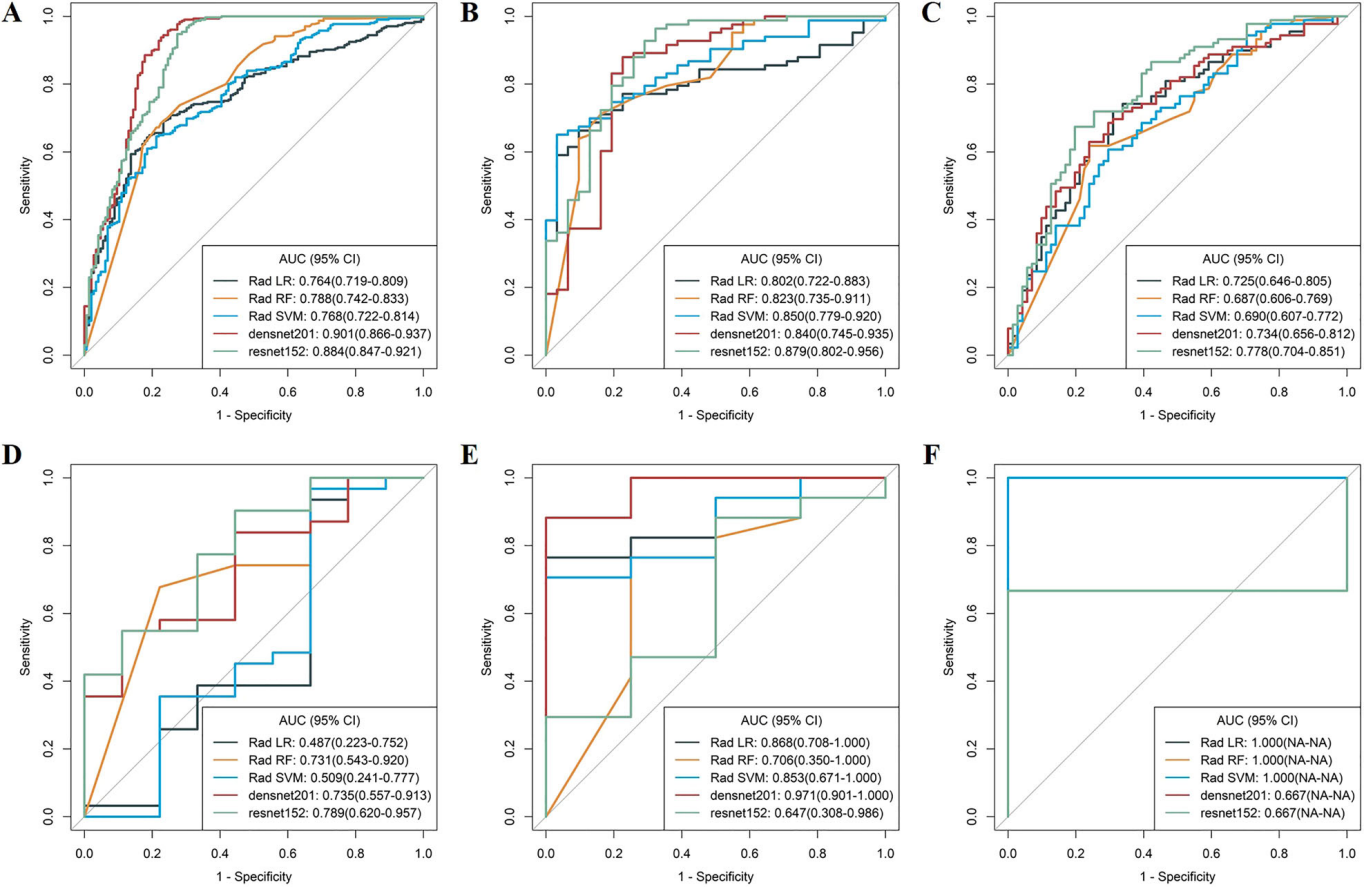
Subgroup analysis of lesion size. Diagnostic performance in training set (**A**), internal validation set (**B**), and external test set (**C**) when HMs and CRLMs are 10–30 mm in size. Diagnostic performance in training set (**D**), internal validation set (**E**), and external test set (**F**) when HMs and CRLMs are ≤ 10 mm

The 10–30 mm group had 589 lesions, including 320 CRLMs, 131 HMs in the training and validation set, 74 CRLMs and 64 HMs in the test set. The subcentimeter group had 207 lesions with 116 CRLMs and 59 HMs in the training and validation set, 24 CRLMs and 8 HMs in the test set. The results of the subgroup analysis demonstrated that when lesion diameter was 10–30 mm, the RN152 model achieved AUCs of 0.884 (95% CI: 0.847–0.921), 0.879 (95% CI: 0.802–0.956), and 0.778 (95% CI: 0.704–0.851) in distinguishing CRLMs from HMs within the training, validation and test set, respectively. When evaluating subcentimetre lesions, the RN152 model demonstrated AUCs of 0.789 (95% CI: 0.620–0.957), 0.647 (95% CI: 0.308–0.986), and 0.667 in differentiating between CRLMs and HMs within the training, validation and test sets, respectively (Fig. 5).

### Performance of DL assistance in diagnosing CRLMs and HMs

The AUCs, sensitivity, and specificity for subjectively distinguishing CRLMs and HMs by five readers with different diagnostic experiences were 0.828 (95% CI: 0.798–0.859), 0.769 (95% CI: 0.724–0.815), and 0.719 (95% CI: 0.673–0.766), respectively. With the DL assistance, AUCs, sensitivity, and specificity for diagnosing CRLMs and HMs were 0.857 (95% CI: 0.828–0.887), 0.755 (95% CI: 0.699–0.810), and 0.806 (95% CI: 0.754–0.857), respectively (Table 5). Overall, DL contributes to the diagnostic capability of radiologists in characterising CRLMs and HMs. The subgroup analysis demonstrated that DL could assist radiologists with varying diagnostic experience in improving the AUCs for differentiating 10–30 mm CRLMs from HMs from 0.851 (95% CI: 0.821–0.880) to 0.879 (95% CI: 0.853–0.906) (*p* = 0.015). The DL model can enhance the confidence of junior radiologists in diagnosing CRLMs and HMs in patients with CRC (Fig. S2). For subcentimeter CRLMs and HMs, the AUCs for the radiologists and the DL-assisted diagnosis were 0.742 (95% CI: 0.669–0.814) and 0.763 (95% CI: 0.681–0.845) (*p* = 0.558), respectively (Table 5).

**Table 5:** Subgroup analysis of the multi-reader subjective diagnosis and DL-assisted diagnosis based on hepatic lesion size

Subgroup		AUC		Sensitivity		Specificity	
		≤ 10 mm	10 mm–30 mm	≤ 10 mm	10 mm–30 mm	≤ 10 mm	10 mm–30 mm
Subjective	Reader 1^a^	0.763	0.871	0.799	0.823	0.603	0.773
	Reader 2^a^	0.768	0.834	0.719	0.752	0.662	0.753
	Reader 3^b^	0.767	0.855	0.755	0.797	0.603	0.794
	Reader 4^c^	0.679	0.841	0.683	0.749	0.632	0.716
	Reader 5^c^	0.734	0.852	0.734	0.780	0.588	0.742
	Pooled (95% CI)	0.742 (0.669–0.814)	0.851 (0.821–0.880)	0.738 (0.666–0.809)	0.780 (0.734–0.827)	0.618 (0.521–0.714)	0.756 (0.702–0.809)
DL assistance	Reader 1^a^	0.820	0.877	0.791	0.800	0.750	0.825
	Reader 2^a^	0.679	0.865	0.554	0.744	0.618	0.804
	Reader 3^b^	0.778	0.873	0.698	0.792	0.632	0.840
	Reader 4^c^	0.734	0.882	0.540	0.790	0.750	0.892
	Reader 5^c^	0.804	0.900	0.748	0.805	0.750	0.856
	Pooled (95% CI)	0.763 (0.681–0.845)	0.879 (0.853–0.906)	0.666 (0.528–0.805)	0.786 (0.745–0.828)	0.700 (0.593–0.807)	0.843 (0.796–0.891)
Difference	ANOVA global test *p* value^*^	0.558	** 0.015**	0.123	0.692	0.108	** 0.019**

The bold text in the table is intended to quickly pinpoint the specific locations where statistically significant differences occur

*CI* confidence interval

^*^ Single-factor analysis of variance

^a^ Reader 1 and a reader 2 were board-certified abdominal radiologists

^b^ Reader 3 was a six-year resident

^c^ Reader 4 and reader 5 were three-year residents

## Discussion

We developed a DL model that performs automated segmentation and classification of CRLMs and HMs on portal venous phase CT, and compared its diagnostic performance against that of radiologists. For 10–30 mm CRLMs and HMs, the automated segmentation achieved a dice coefficient of 0.861. Compared with the subjective evaluation of radiologists, our results found that DL could improve the diagnostic performance of CRLMs and HMs at 10–30 mm (AUC: 0.879 vs 0.851, *p* = 0.015), but could not assist the differential diagnostic performance of subcentimeter CRLMs and HMs.

The Totalsegmentator achieves precise segmentation of CRLMs and HMs; however, suboptimal segmentation accuracy was observed for subcentimetre CRLMs and HMs. Chlebus et al [24] also observed that the U-Net demonstrated inferior segmentation performance for subcentimeter hepatic lesions compared to those measuring ≥ 10 mm. In reality, the segmentation of subcentimeter hepatic lesions using CECT images in conjunction with U-Net remains challenging [25–27]. Balaguer-Montero et al [28] discovered in their system for automatic liver tumor segmentation and detection that performance declined in the segmentation and identification of subcentimeter liver lesions, though specific quantitative data on segmentation accuracy were not provided. The TotalSegmentator achieved a suboptimal dice coefficient of 0.692 for subcentimeter CRLMs and HMs. The suboptimal segmentation performance for subcentimeter lesions can be primarily attributed to the inherent technological limitations of CT imaging. The partial volume effect, a fundamental physical phenomenon, blurs the boundaries of small structures, leading to indistinct imaging features and significant apparent morphological and density variations in these lesions [29]. These intrinsic imaging challenges create a feature-poor environment for DL models. DL models have an increased data dependency, requiring substantially larger and more diverse training datasets to learn robust representations from such ambiguous patterns.

How to reliably differentiate CRLMs from HMs in CRC patients based on the segmentation regions. This study employed radiomics and DL architectures to differentiate between CRLMs and HMs, with comparative analyses revealing that DN201 and RN152 models in the external test dataset demonstrated optimal diagnostic performance, achieving AUCs of 0.735 and 0.776, respectively. Bae et al [9] developed a CECT radiomics model that demonstrated commendable diagnostic efficacy in differentiating LMs, hepatic cysts, and HMs. However, its performance remained inferior to radiologists’ subjective evaluations. The radiomics model developed in our study demonstrated AUCs ranging from 0.701 to 0.741 for differentiating CRLMs from HMs in the external validation cohort, while exhibiting comparatively lower diagnostic efficacy than DL- based architectures. Furthermore, studies utilising dual-energy CT have demonstrated that the spectral curve slope can effectively differentiate between LMs and HMs [30], though this differentiation is contingent upon the selection of VOIs within the lesion. The distinction between CRLMs and HMs can be delineated through contrast enhancement patterns, but small nodules are prone to losing their inherent imaging characteristics [31]. Our developed DL model demonstrated proficiency in discriminating between 10 and 30 mm CRLMs and HMs; however, its performance was suboptimal for subcentimetre lesions. In addition, approximately 20% of CRLMs and HMs were pathologically confirmed, a limitation inherent to the retrospective study design. The characterization of liver lesions based on cross-sectional imaging and follow-up served to robustly demonstrate the value of DL [9, 18]. While the observed decline in the RN152 model’s AUC from 0.858 internally to 0.776 externally indicates a need for improved generalisability, it is noteworthy that the performance on the external test set remains superior to the radiomics models and approaches the baseline performance of junior radiologists. Future validation with multicenter, large-sample datasets is required to assess its applicability, for which prospective, real-world clinical validation is indispensable and likely to determine its potential for clinical implementation.

When implementing DL-assisted diagnosis of CRLMs and HMs, the DL models demonstrated enhanced diagnostic performance for junior radiologists in evaluating 10–30 mm lesions, whereas no statistically significant improvement was observed in diagnostic augmentation for subcentimeter lesions. Consequently, when characterization of subcentimeter hepatic lesions proves challenging in CRC patients, MRI should be employed as the supplementary imaging modality – an approach endorsed within current clinical research [32–35]. A multicenter, large-scale study on AI-assisted automated diagnosis of hepatic lesions has revealed that AI-augmented diagnostic systems can significantly enhance radiologists’ diagnostic efficacy in distinguishing between benign and malignant hepatic lesions [18]. However, the study did not conduct subgroup analysis regarding the diagnostic efficacy of AI-assisted systems in evaluating subcentimeter hepatic lesions. Whilst AI-assisted diagnostic performance for subcentimeter hepatic lesions in CRC patients remains suboptimal, this finding underscores the clinical necessity for repeat MR scanning. A prospective cohort study further recommended that CRC patients with hepatic nodules should undergo additional MR scanning to facilitate early lesion characterization [36]. A meta-analysis further substantiated that MR imaging demonstrates superior sensitivity in both detection and diagnosis of LMs, with the adjunctive use of MRI following CECT impacting clinical management in 16.8% of cases [37]. Based on the results of automatic segmentation and classification of subcentimeter CRLMs and HMs, we also contend that the presence of any indeterminate subcentimeter hepatic lesions on CECT images in CRC patients should warrant an additional MRI scan to assess the likelihood of CRLMs.

The DL model we developed has the potential to be integrated into clinical platforms. When a hypoenhancing nodule, particularly one suspected to be a CRLM or HM, is identified in a CRC patient, the model can be manually activated. It would then provide a differential diagnosis to assist the radiologist, with its output serving as a reference to inform their final comprehensive assessment. Our study has some limitations. First, the primary objective of this study was to explore an end-to-end diagnostic solution for CRLMs and HMs in CRC patients. Consequently, we did not benchmark the accuracy of our method against other automatic segmentation networks, which may result in the generalisability of the automated segmentation outcomes being confined to the scope of this research. Second, radiologists’ subjective diagnosis of CRLMs and HMs is inherently prone to bias. Because, upon identifying a hepatic lesion as metastasis, there is a tendency to categorise other lesions as metastases, particularly in the context of subcentimeter hepatic lesions. Third, the classification of hepatic lesions in CRC patients remains limited in scope, as these lesions extend beyond CRLMs and HMs to encompass entities such as cysts, etc. Fourth, only approximately 20% of the CRLM and HMs patients had pathological confirmation. It does not undermine our primary findings, as we conducted rigorous patient follow-up with cross-sectional imaging comparisons to verify whether the lesions were CRLMs or HMs. Finally, for AI-related studies, the sample size in this research is still insufficient.

## Conclusion

The DL model can assist radiologists in distinguishing 10–30 mm CRLMs from HMs in patients with CRC. However, the auxiliary diagnostic value of DL for subcentimeter CRLMs and HMs lesions has not been effectively validated, and such CRC patients may warrant further MR scans.

## Supplementary information

Supplementary information

## Data Availability

Given that the raw image annotation data amounts to approximately 600 G, should you require the original image annotation data, please contact our team via email at zhangguojin@med.uestc.edu.cn.

## Abbreviations

CECT: Contrast-enhanced computed tomography
CRC: Colorectal cancer
CRLMs: Colorectal cancer liver metastasis
HMs: Hemangiomas
DL: Deep learning
LMs: Liver metastases
LR: Logistic regression
MRI: Magnetic resonance imaging
RF: Random forest
SVM: Support vector machine
