# Supplementary material for: Deep learning in differentiating the colorectal cancer combined with hepatic enhancing nodules: liver metastases vs hemangiomas
Source: Insights Imaging. 2026 Jan 26;17:24. doi: 10.1186/s13244-025-02192-2 (PMC12835484; doi:10.1186/s13244-025-02192-2)
Supplement: Supplementary file 1 — Supplementary information [file 13244_2025_2192_MOESM1_ESM.pdf]

Deep Learning in Differentiating the Colorectal Cancer Combined with Hepatic Enhancing Nodules: Liver Metastases versus Haemangiomas

ELECTRONIC SUPPLEMENTARY MATERIAL

Materials and Methods

CECT image acquisition and Procedures

The enhanced CT data acquisition equipment included: GE Discovery 750 HD, GE Revolution CT (General Electric Company, USA) , Philips Brilliance iCT , Philips Incisive CT, Philips IQon - Spectral CT (Philips AG, Netherlands), SIEMENS SOMATOM Definition Flash CT, and SIEMENS SOMATOM Force CT (Siemens AG, Germany). Prior to enhanced CT examinations, all patients were instructed to fast and consume 500 to 1000 mL of water to distend the gastric lumen. The scanning range extended from the diaphragmatic dome to the pelvic inlet or symphysis pubis level. Conventional spiral scanning parameters are listed in *Table S2, S3*. For CECT scanning, iodinated contrast agent ((iohexol 350 mg/mL, GE HealthCare, China) was administered via high-pressure injection through the antecubital vein at a flow rate of 2.5 to 3.5 mL/s, with a dosage of 1.0 mL per kilogram of body weight. CECT scanning parameters are listed in *Table S3*.

Image Standardization, Segmentation, and Post-processing

Prior to analysis, all CECT scans underwent a standardization procedure to ensure consistent voxel spacing and intensity ranges across the different datasets. Automated liver segmentation was then performed using TotalSegmentator , a deep learning-based framework built upon the nnU-Net architecture. Following this automated step, a two-stage post-processing and quality control protocol was implemented:

1. Visual Quality Assurance: All liver masks generated by TotalSegmentator were subjected to a rigorous quality check by experienced radiologists to identify any potential inaccuracies or artifacts.

2. Manual Correction: In cases where the automated segmentation did not meet the required quality standards (e.g., leakage into adjacent structures or incomplete liver coverage), manual adjustments were performed. This correction was done using ITK-SNAP (version 4.2.0). This meticulous post-processing ensured the accuracy and reliability of the final liver masks before their use in subsequent analyses.

For the conventional radiomics analysis, feature extraction was guided by the Image Biomarker Standardization Initiative (IBSI). The specific preprocessing parameters for this step were implemented using the PyRadiomics library in Python (version 3.8.0). The key parameters included:

- Interpolator Algorithm: A B-Spline interpolator (sitkBSpline) was used for image resampling.
- Resampling: The images were resampled to a uniform voxel spacing of [2, 2, 2] mm.
- Discretization: A fixed bin width of 25 was used for gray-level discretization.

All statistical analyses were performed using R software (version 4.2.0).

Liver automatic segmentation

The CECT scans of the abdomen were first standardized to ensure consistent voxel spacing and intensity ranges across all datasets. TotalSegmentator was then applied to each pre-processed CT volume, generating a 3D binary mask corresponding to the liver region. Minor post-processing was performed to refine the segmentation boundaries and remove any artifacts, resulting in accurate liver masks for subsequent analyses. Following automated segmentation, all liver masks generated were subjected to rigorous quality assurance by experienced radiologists. This step involved visual inspection of the segmented liver regions to identify and correct any inaccuracies or artifacts introduced during the automated process. In cases where the automatic segmentation did not meet the required standards, manual adjustments were performed using ITK-SNAP software to ensure the accuracy and reliability of the liver extraction. The validated liver masks were then utilized for subsequent analyses, including volumetric measurements, lesion detection, and characterization of liver metastases. The automated and validated segmentation process facilitated the efficient handling of large datasets, enabling comprehensive and high-throughput analysis essential for the differentiation of CRLMs.

Lesions automatic segmentation

In another significant part of our study, we focused on the precise segmentation of lesions within the liver. This work built upon our previous efforts using TotalSegmentator for whole-liver segmentation but was conducted as an independent experimental process. We initially utilised the liver segmentation results obtained from TotalSegmentator as a starting point. Based on these outcomes, we extracted and cropped the individual liver region images. This step not only reduced the computational complexity of subsequent processing but also allowed us to concentrate on the most relevant anatomical structures. After acquiring these cropped liver images, we employed the nnUNet framework to train a dedicated liver lesion segmentation model. By training nnU-Net on these pre-processed liver images, we developed a highly specialized model capable of accurately identifying and segmenting various lesions within the liver, such as tumors. This organ-focused

approach enabled the model to learn more subtle features, thereby enhancing the accuracy of lesion detection and segmentation. This independent experiment not only complemented our previous whole-liver segmentation work but also provided an important tool for the precise diagnosis and quantitative analysis of liver diseases. By combining large-scale organ segmentation with refined lesion identification, our research opens new avenues for the automated analysis of liver imaging, with the potential to significantly improve the efficiency and accuracy of clinical diagnoses.

After obtaining the segmentation results from nnU-Net, we implemented a post-processing step to identify and separate individual isolated lesions. This was achieved through connected component analysis, where each spatially disconnected region in the segmentation mask was labeled as a distinct lesion. This approach allowed us to maintain the benefits of whole-liver lesion segmentation during the nnUNet phase while still enabling analysis of individual lesions in subsequent stages of our study.

Conventional radiomic features extraction

Feature extraction followed the Image Biomarker Standardization Initiative (IBSI) guideline in this study. Parameters for extracting features include: normalize: true; normalizeScale: 100; interpolator: ‘sitkBSpline’; resampledPixelSpacing: [2, 2, 2]; binWidth: 25; voxelArrayShift: 300; lable: 1. These features were extracted from three volume of interest (VOI): CECT portal-venous phase image. These features include shape-based feature (N=13), first order features (N=18), gray level co-occurrence matrix (GLCM) features (N=24), gray level dependence matrix (GLDM) features (N=14), gray level run length matrix (GLRLM) features (N=16), gray level size zone matrix (GLSZM) features (N=16), neighboring gray tone difference matrix (NGTDM) features (N=5). Thus, we extracted 106 features from each VOI and 318 algorithm features from the VOI for each patient. The detailed information can be obtained from (https://github.com/AIM-Harvard/pyradiomics).

The training procedure for the three radiomics-based models—Logistic Regression (LR), Random Forest (RF), and Support Vector Machine (SVM)—began with a crucial feature selection step designed to ensure the stability and reproducibility of our results. From the initial set of extracted radiomics features, we selected only the most robust features for

model building by calculating Intraclass Correlation Coefficients (ICCs) on a re-segmented dataset; features with an ICC greater than 0.75 were retained. Following feature selection, the models were constructed using our Python (https://www.python.org; version 3.8.0) environment. In line with establishing a baseline performance for standard radiomics classifiers, we did not perform an exhaustive hyperparameter search. Instead, the models were implemented using the scikit-learn library with its default hyperparameter settings, a common approach for creating standard baseline models for comparison.

DL model's and Radiomics model construction

ResNet3D

We utilized a three-dimensional residual network (ResNet3D) to effectively capture the spatial characteristics of liver lesions. The ResNet architecture, renowned for its deep layers and residual connections, facilitates the learning of intricate three-dimensional features by mitigating the vanishing gradient problem and enabling the training of deeper networks. These residual connections allow the network to preserve spatial information across layers, enhancing its capacity to model the volumetric structures present in contrast-enhanced CT images.

DenseNet3D

Complementing the ResNet architecture, a three-dimensional densely connected convolutional network (DenseNet3D) was employed to further enhance the model. The DenseNet architecture is characterized by its dense connections between layers, which promote feature reuse and efficient gradient propagation. This facilitates the learning of a richer and more diverse set of features, enabling the model to capture subtle texture variations and complex morphological patterns within hepatic lesions. The dense connectivity ensures that each layer receives direct supervision from the loss function, improving feature propagation and reducing the likelihood of overfitting.

Training Protocol

The training protocol was meticulously designed to ensure robust model performance and generalization. Both ResNet3D and DenseNet3D models were trained in an end-to-end manner using the preprocessed and cropped lesion images. To enhance the models' robustness and generalizability, extensive data augmentation techniques were employed, including random rotations, scaling, and contrast adjustments, which simulated a variety of imaging conditions and lesion orientations. A 5-fold cross-validation strategy was implemented to evaluate the models' performance and prevent overfitting, ensuring that each subset of the data served as a validation set in turn while the remaining subsets were used for training. Additionally, an Gradient-weighted Class Activation Mapping (Grad-CAM) was integrated into the network architectures to enhance interpretability and performance, allowing visualization of the specific regions within the lesions that the models focused on during classification. Optimization was performed using the Adam optimizer with an initial learning rate of 1e-4, and a learning rate scheduler was employed to dynamically adjust the learning rate based on validation performance. The models were trained for a maximum of 500 epochs, with early stopping criteria based on the stabilization of validation loss to prevent overfitting. Furthermore, batch normalization and dropout techniques were incorporated to improve model robustness and facilitate the efficient training of deep neural networks. This comprehensive training approach enabled the accurate differentiation between hepatic metastases and hemangiomas, thereby contributing significantly to the study's objective of enhancing diagnostic precision through advanced deep learning methodologies.

Tables

Table S1

Table S1. Characterisation Methods for Hepatic Nodules.

Hepatic lesion confirmation	CRLMs		HMs	
	＜10 mm	10 -30 mm	＜10 mm	10 -30 mm
**Pathological results**				
Training	2/85 (2.35%)	76/85 (89.41%)	/	7/85 (8.24%)
Internal	/	29/31 (93.55%)	/	2/31 (6.45%)
External	/	44/47 (93.62%)	/	3/47 (6.38%)
**Confirmatory imaging (MRI)**				
Training	17 /337 (5.04%)	168/337 (49.85%)	67/337 (19.88%)	81/337 (24.04%)
Internal	16/88 (18.18%)	39/88 (44.32%)	21/88 (23.86%)	12/88 (13.64%)
External	8/109 (7.34%)	31/109 (28.44%)	34/109 (31.19%)	65/109 (59.63%)
**Confirmatory imaging (PET-CT)**				
Training	4/337 (1.19%)	/		
Internal	/	/	/	
External	1/109 (0.92%)	/		
**Follow-up imaging**				
Training	69/69 (100%)			
Internal	16/16 (100%)	/	/	
External	14/14 (100%)			

Table S2

Table S2. Scanning parameters of three-phase abdominal enhanced CT in different instruments.

Parameter	Philips*	GE*	SIEMENS*
Tube voltage, kVp	120	120	80-150
Tube current, mAs	251	250-500	200-400
Collimator width, mm	80	40	60
Rotation time, s	0.5	0.8	0.25
Screw pitch, mm	1.150: 1	0.984: 1	0.8-1.2
Reconstructed layer thickness, mm	1.25	1.25	1.0-1.5

* Note: GE, including Discovery CT 750 HD and GE Revolution CT. Philips, including iCT 256, Incisive CT, and IQon - Spectral CT. SIEMENS, including SOMATOM Definition Flash and SOMATOM Force.

Table S3

Table S3. Abdominal three-phase enhanced CT scan time.

Enhanced phase	Philips*	GE*	SIEMENS*
Arterial phase, s	25-30	25-30	25-30
Portal venous phase, s	45-60	45-55	45-60
Delayed phase, s	120-150	90-120	97-99.5

* Note: GE, including Discovery CT 750 HD and GE Revolution CT. Philips, including iCT 256, Incisive CT, and IQon - Spectral CT. SIEMENS, including SOMATOM Definition Flash and SOMATOM Force.

Table S4

Table S4. Subgroup analysis of nnU-Net automatic segmentation results of different liver lesion sizes.

Subgroup	≤ 10 mm (N=207)	10mm- 30 mm (N=589)	*P* Vaule
Dice	0.692 ± 0.099	0.861 ± 0.033	＜0.001
Jaccard	0.537 ± 0.107	0.758 ± 0.051	＜0.001
Sensitivity	0.662 ± 0.173	0.859 ± 0.065	＜0.001
Specificity	0.999 ± 0.001	0.999 ± 0.001	＜0.001
Labe_volume	1295.084 ± 1463.393	3649.549 ± 6241.768	＜0.001
Pred_volume	1380.283 ± 2145.530	3653.710 ±6217.854	＜0.001

Table S5

Table S5. Delong test for the five models on the training set, internal validation set, and external test set.

Moel	Training set				Internal validation set				External test set			
	≤ 10mm		10 mm- 30 mm		≤ 10mm		10 mm- 30 mm		≤ 10mm		10 mm- 30 mm	
	Z value	*P* value	*Z* value	*P* value	*Z* value	*P* value	*Z* value	*P* value	*Z* value	*P* value	*Z* value	*P* value
Rad LR *vs.* Rad RF	-2.143	**0.032**	-1.774	0.076	1.147	0.251	-0.691	0.489	NA		1.692	0.091
Rad LR *vs.* Rad SVM	-0.679	0.497	-0.485	0.627	0.384	0.701	-3.189	**0.001**			2.677	0.007
Rad LR *vs.* Densnet201	-1.749	**0.080**	-5.257	<**0.001**	-1.040	0.298	-0.648	0.517			-0.182	0.855
Rad LR *vs.* Desnet152	-2.449	**0.014**	-4.261	<**0.001**	1.074	0.283	-1.463	0.144			-1.189	0.234
Rad RF *vs.* Rad SVM	1.999	**0.046**	1.966	**0.049**	-1.201	0.230	-1.174	0.240			-0.151	0.880
Rad RF *vs.* Densnet201	-0.026	0.979	-4.341	<**0.001**	-1.362	0.173	-0.307	0.759			-0.984	0.325
Rad RF *vs.* Resnet152	-0.422	0.673	-3.419	**0.001**	0.199	0.842	-1.083	0.279			-2.070	**0.038**
Rad SVM *vs.* Densnet201	-1.574	0.116	-5.029	<**0.001**	-1.088	0.276	0.178	0.859			-0.918	0.358
Rad SVM *vs.* Resnet152	-2.289	**0.022**	-4.140	<**0.001**	1.005	0.315	-0.641	0.522			-2.004	**0.045**
Densnet201 *vs.* Resnet152	-0.705	0.481	0.862	0.388	1.931	0.053	-0.902	0.367			-1.306	0.191

Note. LR, Logistic regression. RF, Random Forest. SVM, Support vector machine.

Table S6

Table S6. The distribution across data splits is summarised below

Scanner	Overall (N=796)	Training (n = 491)	Internal validation (n = 135)	External test (n = 170)	***P***
GE Discovery CT750 HD	298 (37.44%)	247 (50.31%)	43 (31.85%)	8 (4.71%)	<0.001
GE Revolution CT	154 (19.35%)	130 (26.48%)	24 (17.78%)	0 (0%)	<0.001
Philips iCT 256	135 (16.96%)	81 (16.49%)	54 (40.00%)	0 (0%)	<0.001
Philips Incisive CT	6 (0.75%)	0 (0%)	0 (0%)	6 (3.53%)	<0.001
Philips IQon - Spectral CT	142 (17.84%)	0 (0%)	0 (0%)	142 (83.53%)	<0.001
SIEMENS SOMATOM Definition Flash	5 (0.63%)	0 (0%)	0 (0%)	5 (2.94%)	<0.001
SIEMENS SOMATOM Force	56 (7.04%)	33 (6.72%)	14 (10.37%)	9 (5.29%)	0.230

Note: Three different colours each represent all CECT scanner models from three distinct manufacturers.

Table S7

Table S7 demonstrates the performance of contrast-enhanced CT scanners from three different manufacturers in differentiating CRLMs from HMs.

seed	method	feature_nums	Cutoff	train_GE_auc	train_GE_acc	train_GE_sen	train_GE_spe	train_GE_ppv	train_GE_npv
1	Rad LR	2	0.73	0.762(0.711-0.812)	0.698(0.649-0.744)	0.643(0.473-0.737)	0.815(0.706-0.899)	0.883(0.847-0.896)	0.513(0.477-0.538)
1	Rad RF	2	0.89	0.803(0.755-0.852)	0.716(0.668-0.761)	0.694(0.601-0.782)	0.765(0.691-0.861)	0.865(0.847-0.878)	0.535(0.510-0.565)
1	Rad SVM	2	0.73	0.769(0.717-0.820)	0.690(0.640-0.736)	0.647(0.531-0.733)	0.782(0.672-0.866)	0.865(0.840-0.879)	0.505(0.468-0.531)
1	DN201	0	1.00	0.886(0.845-0.927)	0.849(0.809-0.883)	0.876(0.639-0.934)	0.790(0.689-0.866)	0.900(0.868-0.906)	0.746(0.719-0.763)
1	RN152	0	0.99	0.848(0.802-0.894)	0.854(0.814-0.888)	0.953(0.818-1.000)	0.639(0.546-0.731)	0.851(0.831-0.857)	0.864(0.844-0.879)
				train_Philips_auc	train_Philips_acc	train_Philips_sen	train_Philips_spe	train_Philips_ppv	train_Philips_npv
1	Rad LR	2	0.73	0.617(0.477-0.757)	0.630(0.515-0.734)	0.645(0.386-0.806)	0.579(0.263-0.789)	0.833(0.750-0.862)	0.333(0.185-0.405)
1	Rad RF	2	0.89	0.650(0.504-0.796)	0.654(0.540-0.757)	0.677(0.398-0.855)	0.579(0.316-0.789)	0.840(0.755-0.869)	0.355(0.231-0.429)
1	Rad SVM	2	0.73	0.647(0.504-0.790)	0.654(0.540-0.757)	0.677(0.434-0.808)	0.579(0.263-0.842)	0.840(0.771-0.862)	0.355(0.200-0.444)
1	DNt201	0	1.00	0.895(0.791-0.998)	0.901(0.815-0.956)	0.935(0.371-1.000)	0.789(0.632-0.947)	0.935(0.852-0.939)	0.789(0.750-0.818)
1	RN152	0	0.99	0.935(0.850-1.000)	0.951(0.878-0.986)	1.000(0.709-1.000)	0.789(0.579-0.949)	0.939(0.917-0.939)	1.000(1.000-1.000)
				train_SIEMENS_auc	train_SIEMENS_acc	train_SIEMENS_sen	train_SIEMENS_spe	train_SIEMENS_ppv	train_SIEMENS_npv
1	Rad LR	2	0.73	0.838(0.699-0.977)	0.818(0.645-0.930)	0.812(0.375-0.938)	0.824(0.353-1.000)	0.812(0.667-0.833)	0.824(0.667-0.850)
1	Rad RF	2	0.89	0.812(0.664-0.961)	0.818(0.645-0.930)	0.812(0.219-0.938)	0.824(0.323-0.941)	0.812(0.538-0.833)	0.824(0.647-0.842)
1	Rad SVM	2	0.73	0.809(0.661-0.957)	0.758(0.577-0.889)	0.688(0.188-0.938)	0.824(0.471-0.943)	0.786(0.500-0.833)	0.737(0.615-0.762)
1	DN201	0	1.00	0.926(0.833-1.000)	0.818(0.645-0.930)	0.688(0.188-1.000)	0.941(0.706-1.000)	0.917(0.750-0.941)	0.762(0.706-0.773)
1	RN152	0	0.99	0.941(0.845-1.000)	0.939(0.798-0.993)	0.938(0.062-1.000)	0.941(0.647-1.000)	0.938(0.500-0.941)	0.941(0.917-0.944)
				val_GE_auc	val_GE_acc	val_GE_sen	val_GE_spe	val_GE_ppv	val_GE_npv
1	Rad LR	2	0.73	0.788(0.674-0.902)	0.672(0.546-0.782)	0.608(0.333-0.785)	0.875(0.623-1.000)	0.939(0.895-0.952)	0.412(0.333-0.444)
1	Rad RF	2	0.89	0.792(0.657-0.927)	0.657(0.531-0.768)	0.627(0.439-0.863)	0.750(0.500-1.000)	0.889(0.848-0.917)	0.387(0.296-0.457)

1	Rad SVM	2	0.73	0.819(0.709-0.928)	0.687(0.562-0.794)	0.647(0.490-0.902)	0.812(0.500-1.000)	0.917(0.893-0.939)	0.419(0.308-0.471)
1	DN201	0	1.00	0.766(0.605-0.927)	0.851(0.743-0.926)	0.902(0.255-0.961)	0.688(0.250-0.875)	0.902(0.722-0.907)	0.688(0.444-0.737)
1	RN152	0	0.99	0.826(0.692-0.960)	0.881(0.778-0.947)	0.941(0.392-1.000)	0.688(0.375-0.812)	0.906(0.800-0.911)	0.786(0.667-0.812)
				val_Philips_auc	val_Philips_acc	val_Philips_sen	val_Philips_spe	val_Philips_ppv	val_Philips_npv
1	Rad LR	2	0.73	0.792(0.667-0.916)	0.685(0.544-0.805)	0.673(0.551-0.878)	0.800(0.595-1.000)	0.971(0.964-0.977)	0.200(0.157-0.238)
1	Rad RF	2	0.89	0.720(0.466-0.975)	0.722(0.584-0.835)	0.714(0.133-0.878)	0.800(0.200-1.000)	0.972(0.867-0.977)	0.222(0.067-0.263)
1	Rad SVM	2	0.73	0.824(0.707-0.942)	0.741(0.603-0.850)	0.714(0.592-0.878)	1.000(0.400-1.000)	1.000(1.000-1.000)	0.263(0.125-0.263)
1	DN201	0	1.00	0.927(0.841-1.000)	0.796(0.665-0.894)	0.796(0.714-1.000)	0.800(0.595-1.000)	0.975(0.972-0.980)	0.286(0.229-0.333)
1	RN152	0	0.99	0.853(0.602-1.000)	0.926(0.821-0.979)	0.959(0.285-1.000)	0.600(0.000-1.000)	0.959(0.875-0.961)	0.600(0.000-0.714)
				val_SIEMENS_auc	val_SIEMENS_acc	val_SIEMENS_sen	val_SIEMENS_spe	val_SIEMENS_ppv	val_SIEMENS_npv
1	Rad LR	2	0.73	NaN	0.929(0.661-0.998)	NA	0.929	NA	NA
1	Rad RF	2	0.89	NaN	0.929(0.661-0.998)	NA	0.929	NA	NA
1	Rad SVM	2	0.73	NaN	0.929(0.661-0.998)	NA	0.929	NA	NA
1	DN201	0	1.00	NaN	0.857(0.572-0.982)	NA	0.857	NA	NA
1	RN152	0	0.99	NaN	0.643(0.351-0.872)	NA	0.643	NA	NA
				test_GE_auc	test_GE_acc	test_GE_sen	test_GE_spe	test_GE_ppv	test_GE_npv
1	Rad LR	2	0.73	NaN	0.875(0.473-0.997)	NA	0.875	NA	NA
1	Rad RF	2	0.89	NaN	0.875(0.473-0.997)	NA	0.875	NA	NA
1	Rad SVM	2	0.73	NaN	0.875(0.473-0.997)	NA	0.875	NA	NA
1	DN201	0	1.00	NaN	0.750(0.349-0.968)	NA	0.75	NA	NA
1	RN152	0	0.99	NaN	0.750(0.349-0.968)	NA	0.75	NA	NA
				test_Philips_auc	test_Philips_acc	test_Philips_sen	test_Philips_spe	test_Philips_ppv	test_Philips_npv

1	Rad LR	2	0.73	0.723(0.637-0.808)	0.662(0.580-0.738)	0.606(0.404-0.798)	0.759(0.574-0.870)	0.814(0.745-0.852)	0.526(0.456-0.560)
1	Rad RF	2	0.89	0.693(0.605-0.781)	0.682(0.601-0.756)	0.649(0.333-0.739)	0.741(0.485-0.834)	0.813(0.691-0.832)	0.548(0.443-0.577)
1	Rad SVM	2	0.73	0.693(0.604-0.782)	0.635(0.552-0.713)	0.596(0.404-0.755)	0.704(0.537-0.833)	0.778(0.704-0.816)	0.500(0.433-0.542)
1	DN201	0	1.00	0.724(0.638-0.809)	0.655(0.573-0.732)	0.638(0.447-0.809)	0.685(0.556-0.834)	0.779(0.712-0.817)	0.521(0.469-0.570)
1	RN152	0	0.99	0.766(0.684-0.848)	0.750(0.672-0.817)	0.872(0.691-0.936)	0.537(0.351-0.667)	0.766(0.722-0.779)	0.707(0.613-0.750)
				test_SIEMENS_auc	test_SIEMENS_acc	test_SIEMENS_sen	test_SIEMENS_spe	test_SIEMENS_ppv	test_SIEMENS_npv
1	Rad LR	2	0.73	0.750(0.479-1.000)	0.714(0.419-0.916)	0.500(0.000-1.000)	0.800(0.498-1.000)	0.500(0.000-0.667)	0.800(0.713-0.833)
1	Rad RF	2	0.89	0.650(0.330-0.970)	0.714(0.419-0.916)	0.500(0.000-1.000)	0.800(0.200-1.000)	0.500(0.000-0.667)	0.800(0.500-0.833)
1	Rad SVM	2	0.73	0.725(0.451-0.999)	0.571(0.289-0.823)	0.000(0.000-1.000)	0.800(1.000-1.000)	0.000(0.000-0.667)	0.667(0.714-0.714)
1	DN201	0	1.00	0.625(0.218-1.000)	0.714(0.419-0.916)	0.250(0.000-0.750)	0.900(0.400-1.000)	0.500(0.000-0.750)	0.750(0.571-0.769)
1	RN152	0	0.99	0.675(0.368-0.982)	0.643(0.351-0.872)	0.500(0.000-1.000)	0.700(0.300-1.000)	0.400(0.000-0.571)	0.778(0.600-0.833)

Note: auc, Area under the curve. acc, Accuracy. ppv, Positive predictive value. npv, Negative predictive value. sen, Sensitivity. spe, Specificity. Rad, Radiomics. LR, Logistic regression. RF, Random Forest.

Table S8

Table S8 presents the performance of the five predictive models in distinguishing between CRLMs and HMs across the training, validation, and test sets.

seed	method	feature_nums	Cutoff	train_auc	train_acc	train_sen	train_spe	train_ppv	train_npv
1	Rad LR	2	0.73	0.750(0.704-0.796)	0.695(0.652-0.735)	0.652(0.506-0.732)	0.787(0.690-0.845)	0.869(0.837-0.882)	0.510(0.478-0.528)
1	Rad RF	2	0.89	0.784(0.740-0.829)	0.713(0.671-0.752)	0.696(0.606-0.768)	0.748(0.665-0.819)	0.857(0.839-0.869)	0.532(0.503-0.555)
1	Rad SVM	2	0.73	0.756(0.709-0.802)	0.688(0.645-0.729)	0.655(0.509-0.720)	0.761(0.658-0.832)	0.856(0.822-0.867)	0.504(0.468-0.527)
1	DN201	0	1.00	0.892(0.857-0.927)	0.855(0.821-0.885)	0.878(0.681-0.938)	0.806(0.729-0.865)	0.908(0.884-0.913)	0.753(0.734-0.766)
1	RN152	0	0.99	0.875(0.838-0.912)	0.876(0.843-0.904)	0.961(0.824-0.997)	0.690(0.606-0.768)	0.871(0.852-0.875)	0.892(0.879-0.902)
				val_auc	val_acc	val_sen	val_spe	val_ppv	val_npv
1	Rad LR	2	0.73	0.814(0.743-0.885)	0.704(0.619-0.779)	0.640(0.540-0.780)	0.886(0.743-1.000)	0.941(0.931-0.951)	0.463(0.419-0.493)
1	Rad RF	2	0.89	0.810(0.724-0.896)	0.711(0.627-0.786)	0.670(0.390-0.815)	0.829(0.686-0.971)	0.918(0.867-0.931)	0.468(0.421-0.507)
1	Rad SVM	2	0.73	0.849(0.785-0.914)	0.733(0.650-0.806)	0.680(0.570-0.811)	0.886(0.657-1.000)	0.944(0.934-0.953)	0.492(0.418-0.522)
1	DN201	0	1.00	0.847(0.759-0.935)	0.830(0.755-0.889)	0.850(0.310-0.950)	0.771(0.629-0.943)	0.914(0.795-0.922)	0.643(0.595-0.688)
1	RN152	0	0.99	0.858(0.781-0.935)	0.874(0.806-0.925)	0.950(0.730-1.000)	0.657(0.429-0.800)	0.888(0.859-0.893)	0.821(0.750-0.848)
				test_auc	test_acc	test_sen	test_spe	test_ppv	test_npv

1	Rad LR	2	0.73	0.741(0.665-0.817)	0.676(0.601-0.746)	0.602(0.388-0.766)	0.778(0.639-0.861)	0.787(0.704-0.824)	0.589(0.541-0.614)
1	Rad RF	2	0.89	0.701(0.622-0.780)	0.694(0.619-0.762)	0.643(0.347-0.725)	0.764(0.444-0.861)	0.787(0.667-0.807)	0.611(0.478-0.639)
1	Rad SVM	2	0.73	0.706(0.627-0.785)	0.641(0.564-0.713)	0.571(0.367-0.735)	0.736(0.597-0.847)	0.747(0.655-0.791)	0.558(0.506-0.592)
1	DN201	0	1.00	0.735(0.658-0.811)	0.665(0.588-0.735)	0.622(0.490-0.765)	0.722(0.597-0.861)	0.753(0.706-0.789)	0.584(0.538-0.626)
1	RN152	0	0.99	0.776(0.703-0.848)	0.741(0.669-0.805)	0.857(0.694-0.939)	0.583(0.416-0.667)	0.737(0.694-0.754)	0.750(0.682-0.774)

Table S9

Table S9 demonstrates the distinguishing efficacy of the five models for sub-centimetre and 10–30 mm CRLMs and HMs across the training, validation, and test sets.

seed	method	feature_nums	Cutoff	train_10_auc	train_10_acc	train_10_sen	train_10_spe	train_10_ppv	train_10_npv
1	Rad LR	2	0.73	0.487(0.223-0.752)	0.650(0.483-0.794)	0.742(0.226-1.000)	0.333(0.111-0.667)	0.793(0.538-0.838)	0.273(0.111-0.429)
1	Rad RF	2	0.89	0.731(0.543-0.920)	0.700(0.535-0.834)	0.806(0.613-1.000)	0.333(0.111-0.889)	0.806(0.760-0.838)	0.333(0.143-0.571)
1	Rad SVM	2	0.73	0.509(0.241-0.777)	0.650(0.483-0.794)	0.742(0.290-1.000)	0.333(0.111-0.667)	0.793(0.600-0.838)	0.273(0.111-0.429)
1	DN201	0	1.00	0.735(0.557-0.913)	0.750(0.588-0.873)	0.806(0.483-1.000)	0.556(0.111-0.889)	0.862(0.789-0.886)	0.455(0.143-0.571)
1	RN152	0	0.99	0.789(0.620-0.957)	0.825(0.672-0.927)	0.903(0.484-1.000)	0.556(0.111-0.889)	0.875(0.789-0.886)	0.625(0.250-0.727)
				train_10_30_auc	train_10_30_acc	train_10_30_sen	train_10_30_spe	train_10_30_ppv	train_10_30_npv
1	Rad LR	2	0.73	0.764(0.719-0.809)	0.698(0.654-0.740)	0.643(0.541-0.705)	0.815(0.699-0.891)	0.879(0.859-0.888)	0.522(0.483-0.544)
1	Rad RF	2	0.89	0.788(0.742-0.833)	0.714(0.670-0.755)	0.685(0.566-0.776)	0.774(0.682-0.843)	0.864(0.839-0.878)	0.541(0.509-0.562)
1	Rad SVM	2	0.73	0.768(0.722-0.814)	0.692(0.647-0.734)	0.646(0.515-0.712)	0.788(0.664-0.843)	0.864(0.835-0.875)	0.516(0.473-0.533)
1	DN201	0	1.00	0.901(0.866-0.937)	0.865(0.830-0.895)	0.885(0.600-0.948)	0.822(0.733-0.870)	0.912(0.876-0.917)	0.774(0.753-0.784)

1	RN152	0	0.99	0.884(0.847-0.921)	0.880(0.847-0.909)	0.967(0.826-1.000)	0.699(0.616-0.788)	0.870(0.851-0.874)	0.911(0.900-0.920)
				val_10_auc	val_10_acc	val_10_sen	val_10_spe	val_10_ppv	val_10_npv
1	Rad LR	2	0.73	0.868(0.708-1.000)	0.762(0.528-0.918)	0.765(0.647-1.000)	0.750(0.250-1.000)	0.929(0.917-0.944)	0.429(0.200-0.500)
1	Rad RF	2	0.89	0.706(0.350-1.000)	0.714(0.478-0.887)	0.765(0.274-1.000)	0.500(0.000-1.000)	0.867(0.699-0.895)	0.333(0.000-0.500)
1	Rad SVM	2	0.73	0.853(0.671-1.000)	0.714(0.478-0.887)	0.765(0.588-1.000)	0.500(0.250-1.000)	0.867(0.833-0.895)	0.333(0.200-0.500)
1	DN201	0	1.00	0.971(0.901-1.000)	0.857(0.637-0.970)	0.882(0.706-1.000)	0.750(0.494-1.000)	0.938(0.923-0.944)	0.600(0.497-0.667)
1	RN152	0	0.99	0.647(0.308-0.986)	0.810(0.581-0.946)	0.882(0.176-1.000)	0.500(0.000-1.000)	0.882(0.600-0.895)	0.500(0.000-0.667)
				val_10_30_auc	val_10_30_acc	val_10_30_sen	val_10_30_spe	val_10_30_ppv	val_10_30_npv
1	Rad LR	2	0.73	0.802(0.722-0.883)	0.693(0.600-0.776)	0.614(0.503-0.807)	0.903(0.774-1.000)	0.944(0.933-0.957)	0.467(0.429-0.492)
1	Rad RF	2	0.89	0.823(0.735-0.911)	0.711(0.618-0.792)	0.651(0.317-0.807)	0.871(0.688-0.969)	0.931(0.868-0.944)	0.482(0.424-0.509)
1	Rad SVM	2	0.73	0.850(0.779-0.920)	0.737(0.646-0.815)	0.663(0.385-0.759)	0.935(0.742-1.000)	0.965(0.941-0.969)	0.509(0.451-0.525)
1	DN201	0	1.00	0.840(0.745-0.935)	0.825(0.742-0.889)	0.843(0.349-0.952)	0.774(0.644-0.935)	0.909(0.805-0.919)	0.649(0.606-0.690)
1	RN152	0	0.99	0.879(0.802-0.956)	0.886(0.813-0.938)	0.964(0.723-1.000)	0.677(0.419-0.807)	0.889(0.857-0.892)	0.875(0.812-0.893)
				test_10_auc	test_10_acc	test_10_sen	test_10_spe	test_10_ppv	test_10_npv

1	Rad LR	2	0.73	1.000(NA-NA)	0.900(0.555-0.997)	0.889(1.000-1.000)	1.000(1.000-1.000)	1.000(1.000-1.000)	0.500(0.500-0.500)
1	Rad RF	2	0.89	1.000(NA-NA)	0.900(0.555-0.997)	0.889(1.000-1.000)	1.000(1.000-1.000)	1.000(1.000-1.000)	0.500(0.500-0.500)
1	Rad SVM	2	0.73	1.000(NA-NA)	0.900(0.555-0.997)	0.889(1.000-1.000)	1.000(1.000-1.000)	1.000(1.000-1.000)	0.500(0.500-0.500)
1	DN201	0	1.00	0.667(NA-NA)	0.600(0.262-0.878)	0.556(0.333-1.000)	1.000(0.000-1.000)	1.000(1.000-1.000)	0.200(0.000-0.200)
1	RN152	0	0.99	0.667(NA-NA)	0.900(0.555-0.997)	1.000(1.000-1.000)	0.000(0.000-0.000)	0.900(0.900-0.900)	NaN(NaN-NaN)
				test_10_30_auc	test_10_30_acc	test_10_30_sen	test_10_30_spe	test_10_30_ppv	test_10_30_npv
1	Rad LR	2	0.73	0.725(0.646-0.805)	0.662(0.584-0.735)	0.573(0.393-0.764)	0.775(0.662-0.873)	0.761(0.686-0.810)	0.591(0.553-0.620)
1	Rad RF	2	0.89	0.687(0.606-0.769)	0.681(0.603-0.753)	0.618(0.327-0.708)	0.761(0.454-0.866)	0.764(0.631-0.788)	0.614(0.487-0.644)
1	Rad SVM	2	0.73	0.690(0.607-0.772)	0.625(0.545-0.700)	0.539(0.326-0.719)	0.732(0.605-0.845)	0.716(0.604-0.771)	0.559(0.512-0.594)
1	DN201	0	1.00	0.734(0.656-0.812)	0.669(0.590-0.741)	0.629(0.505-0.776)	0.718(0.592-0.845)	0.737(0.692-0.775)	0.607(0.560-0.645)
1	RN152	0	0.99	0.778(0.704-0.851)	0.731(0.656-0.798)	0.843(0.674-0.910)	0.592(0.394-0.690)	0.721(0.674-0.736)	0.750(0.666-0.778)

Note: 10, Subcentimeter lesion. 10- 30, 10- 30 mm lesion.

Figure S1

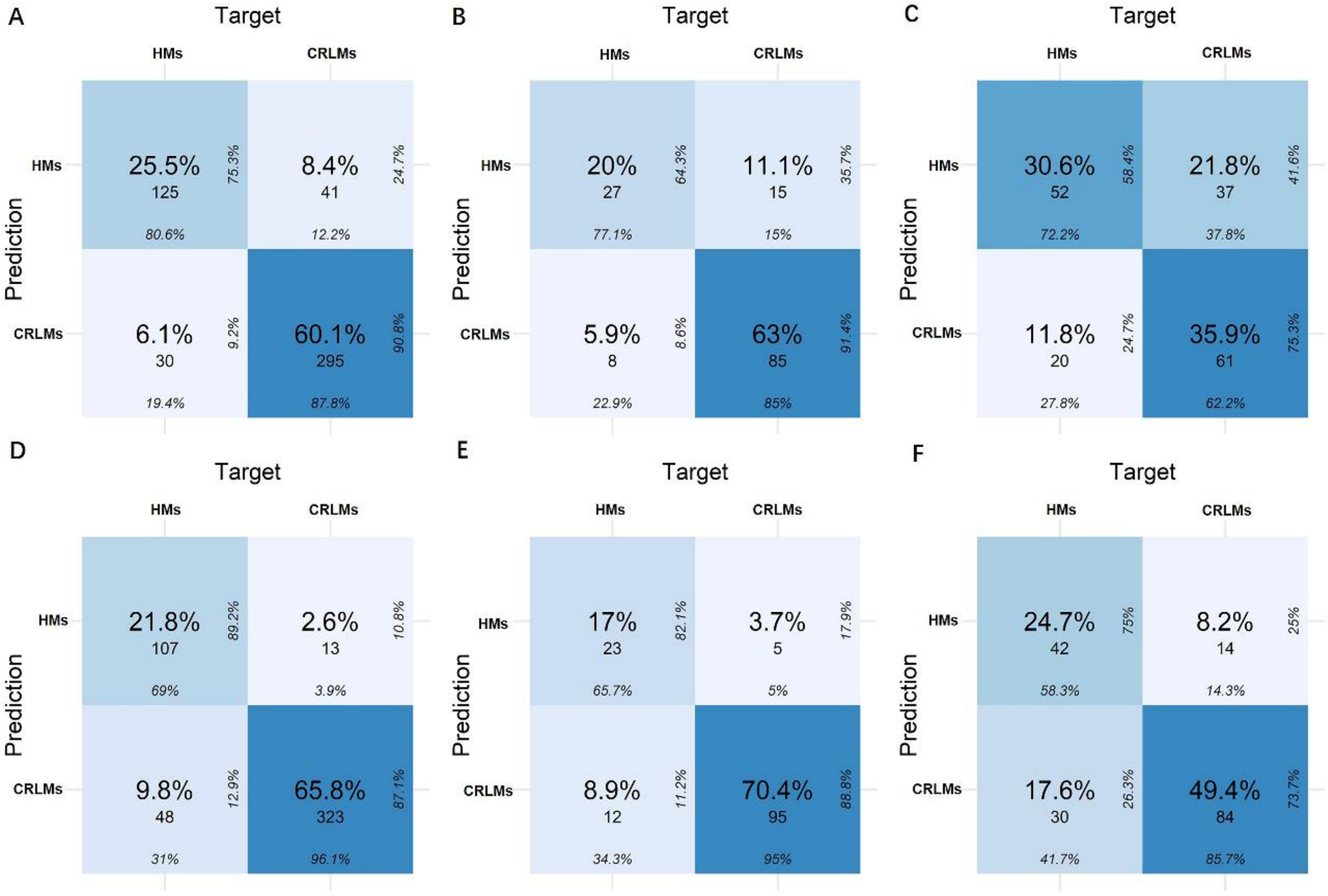

Figure S1. A, B, and C present the confusion matrices for the DN201 model in distinguishing between CRLMs and HMs within the training, validation, and test sets, respectively. D, E, and F display the confusion matrices for the RN152 model classification performance across the datasets.

Figure S2

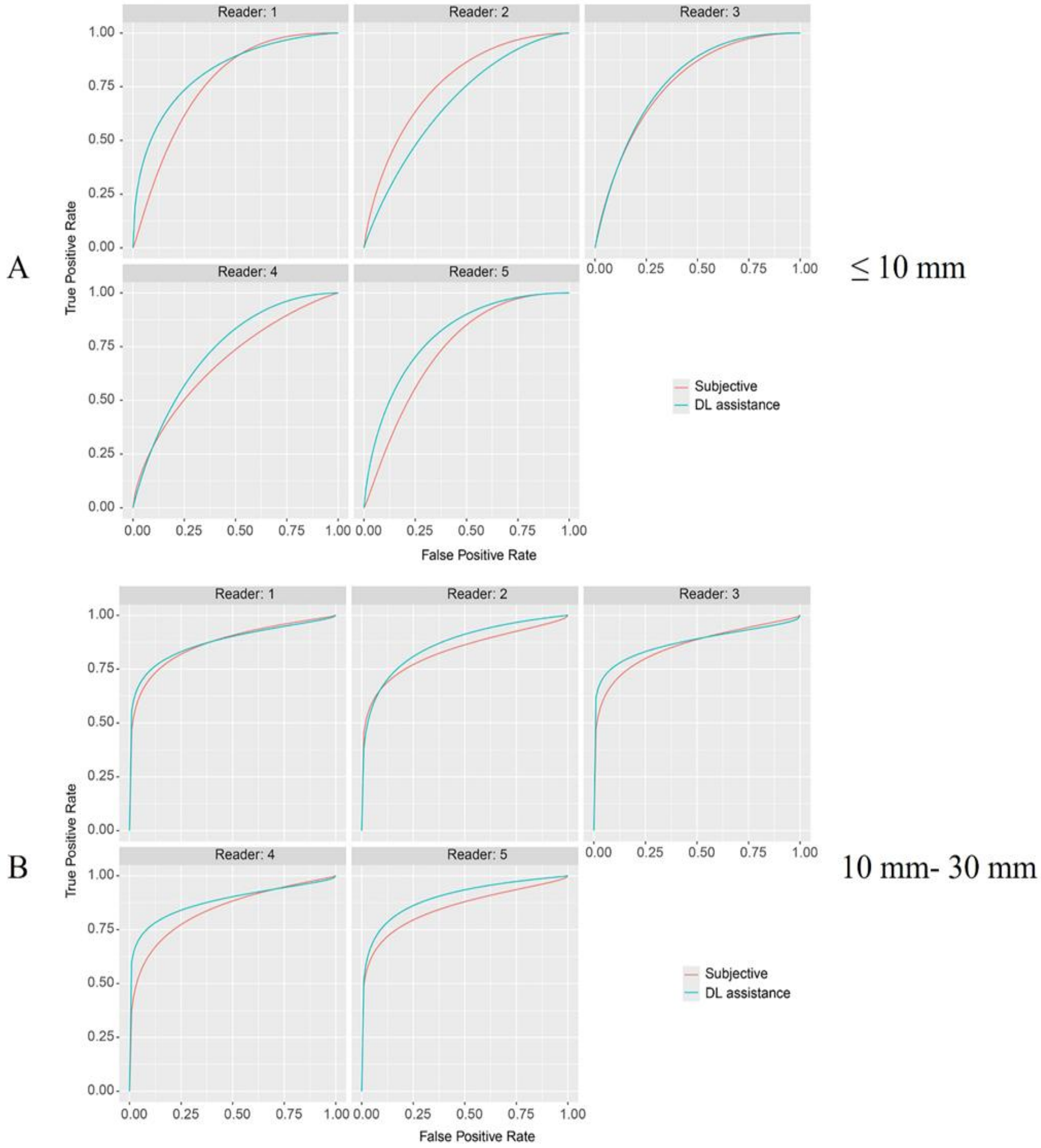

Figure S2. Subgroup analysis of the multi-reader subjective diagnosis and DL-assisted diagnosis CRLMs or HMs based on hepatic lesion size. A. Both CRLMs and HMs were less than or equal to 10mm. B. Both CRLMs and HMs were greater than 10mm and less than or equal to 30mm.
